# Census Bulletin, Nos. 22, 25, 26, 27, 29, 30

**Published:** 1891-03

**Authors:** 


CENSUS BULLETIN. Hon. Robert P. Porter, Superintendent of Census, Washington, D. C.

No. 22 gives statistics of distilled spirits used in the arts, in manufactures, and in medicine. No. 25, statistics of the Indians, from which it appears that the total Indian population of the United States is 249,273. No. 26, statistics of Maryland coal; No. 27, Alabama coal; No. 29, transportation; and No. 30, statistics of the population of Alaska.

